# Depression and Anxiety in Patients with Rare Diseases during the COVID-19 Pandemic

**DOI:** 10.3390/ijerph18063234

**Published:** 2021-03-21

**Authors:** Juan Carlos Sánchez-García, Jonathan Cortés-Martín, Raquel Rodríguez-Blanque, Ana Eugenia Marín-Jiménez, Maria Montiel-Troya, Lourdes Díaz-Rodríguez

**Affiliations:** 1 Research Group CTS1068, Andalusia Research Plan, Junta de Andalucía, JMADOC, School of Nursing, Faculty of Health Sciences, University of Granada, 18071 Granada, Spain; jsangar@ugr.es; 2 Research Group CTS 1068, Andalusia Research Plan, Junta de Andalucía, Hospital Universitario Virgen de las Nieves, 18014 Granada, Spain; jonathan.cortes.martin@gmail.com; 3 Research Group CTS1068, Andalusia Research Plan, Junta de Andalucía, Distrito Sanitario Granada-Metropolitano, 18013 Granada, Spain; 4 Research Group CTS1068, Andalusia Research Plan, Junta de Andalucía, Quantitative Methods for the Economics and Enterprise, Faculty of Economics and Business Sciences, University of Granada, 18071 Granada, Spain; anamarin@ugr.es; 5 Research Group CTS1068, Andalusia Research Plan, Junta de Andalucía, School of Nursing Ceuta Campus, Faculty of Health Sciences, University of Granada, 51001 Ceuta, Spain; mariamontiel@ugr.es; 6 Research Group CTS1068, Andalusia Research Plan, Junta de Andalucía, School of Nursing, Faculty of Health Sciences, University of Granada, 18071 Granada, Spain; cldiaz@ugr.es

**Keywords:** rare disease, depression, anxiety, COVID-19, SARS CoV-2, pandemic

## Abstract

Scientific knowledge on depression and anxiety in patients with rare diseases during the COVID-19 pandemic is scarce; however, it is essential to perform comprehensive management of these patients. The aim of this study was to research how the situation caused by the SARS-CoV-2 pandemic has influenced the lives of patients with rare diseases regarding depression and anxiety. This Spanish study considered a heterogeneous population sample of 86 patients with confirmed diagnosis of different rare diseases. Participants took part in a cross-sectional online study by completing specific questionnaires on the study topic. Depression was measured using the Patient Health Questionnaire (PHQ-9), and the General Anxiety Disorder Scale (GAD-7) was used for evaluating anxiety. Data collection through an online questionnaire allowed for a greater population scope and therefore the inclusion patients of other nationalities in the study sample. Finally, as a general result, this study found that, in the face of the pandemic, anxiety and depression remained at a higher level in this group than in the general population, making these patients a vulnerable population group.

## 1. Introduction

Rare diseases are conditions that are mainly characterized by their low prevalence in the population [1]. They are also known as orphan diseases. In order to be considered a rare disease, it must affect fewer than 1 in 2000 people [2]. There may be as many as 7000 rare diseases, 80% of which are genetic. These diseases are clinically and phenotypically very heterogeneous due to the variability of expression of the mutated gene that causes them. Most of these diseases are chronic and progressive and normally have an important negative impact on the quality of life of the affected patients [1].

In order to fully grasp the complexity of rare diseases, it is necessary to study in depth the different clinical aspects that characterize them, the specific circumstances that surround each disease, and the repercussions they have on patients, their families and their environment, as well as the interactions between the previously mentioned elements [3].

Due to the low number of cases and the specificity of each disease, it is often the case that scientific knowledge, and therefore, the scientific literature on each disease is directly proportional to the number of cases that have been described [4].

This fact has a negative impact on the diagnostic phase, making it hard to establish a definitive diagnosis at early stages. Diagnostic delay [5] hinders clinical management of the disease, leading to a situation of uncertainty and chaos that is harmful for the patient, worsening the symptoms of their disease and increasing the likelihood of development of mental disorders.

On 11 March 2020, the World Health Organization declared the infectious disease caused by the coronavirus discovered in 2019, named COVID-19, a pandemic [6]. Since then, many governments have imposed a series of measures that make isolation and social distancing compulsory. Namely, in Spain [7], the country in which this study was carried out, measures of home confinement were established, and all non-essential services were prohibited, as was free, non-justified circulation. Schools were closed and the use of face masks was made obligatory, as was a social distance of 2 m. These measures remained in place from 14 March to the end of June 2020. Over time, we have seen how the government applied or lifted impositions depending on the state of the COVID-19 pandemic throughout the country. These measures have limited the access to primary medical care, and access to mental health services and other types of support centers has been greatly disrupted.

This pandemic and the public health measures that were established can have serious psychological repercussions on any person. Additionally, patients with rare diseases may be at risk of suffering debilitating psychological symptoms as a direct consequence of the progression of their own disease [3]. Additionally, the development of this pandemic negatively impacts these patients, who have become a vulnerable population group with regard to the issue at hand [8].

There are studies that report the psychological burden and the increase in mental health symptoms in patients with rare diseases [8,9]. The increase in depressive and anxiety syndromes in certain rare diseases is also known [10], but in general, scientific publications on the psychopathology of rare diseases are scarce and, even more so, with regard to how they may relate to the worldwide COVID-19 pandemic.

Depression and anxiety are frequent psychosocial symptoms in chronic diseases [11]. Depression is a frequent mental disorder that is characterized by persistent sadness and a loss of interest in activities that are normally enjoyable, accompanied by an incapability to carry out daily activities. Anxiety is an emotional response that arises in the face of situations that the person perceives or interprets as menacing or dangerous [12].

As no curative treatment exists for most rare diseases [1], it is of interest to study depression and anxiety as related symptoms in a specific context, such as that generated by the COVID-19 pandemic, as the treatment of such symptoms would likely improve the global quality of life of these patients.

The aim of this study was to research how the situation caused by the SARS-CoV-2 pandemic has influenced the lives of patients with rare diseases regarding depression and anxiety.

## 2. Materials and Methods

This study was a cross-sectional online study. Due to the pandemic situation caused by the coronavirus SARS-CoV-2, the whole study was carried out using online questionnaires. This way, it was possible for the study to acquire an international perspective.

The study was approved by the Ethics Committee of the province of Granada. At all times, the study was performed in accordance with the guidelines of the Helsinki Declaration, amended by the 64th General Assembly of the AMM, in Fortaleza, Brazil, in October 2013. Before completing the online questionnaire, participants were requested to give their informed consent online, by means of a box on the form that had to be intentionally ticked in order to continue with the questionnaire.

### 2.1. Participants

Patients were recruited through rare disease patient associations. Information on the study was distributed via mailing lists of rare disease patient associations. An information leaflet with information on the study was attached and the email included a link to an online form specifically designed for this study (see in Supplementary Materials).

A special email address was created for this study, through which patients interested in taking part could contact the research team with any questions.

All patients diagnosed with a rare disease could take part in the study. Diseases with a prevalence of 1 in 2000 people were considered rare diseases and included in the study. If no information regarding prevalence was available, patients were included if their disease is listed on the Orpha.net registry of rare diseases [2].

The minimum age for participation in the study was 18 years of age, with no upper limit. Patients with an unclear diagnosis, with a disease not considered a rare disease, or patients who said that the diagnosis was not given by a doctor were excluded.

The patients who answered “Yes” to the question “Do you have a recent history of symptoms of anxiety or depression in the months prior to the pandemic?” were excluded. Extensive lists of symptoms of anxiety and depression were detailed in the question. The symptoms of anxiety considered include psychological symptoms such as constant worry, tiredness, irritability, and difficulty concentrating or sleeping problems, and physical symptoms such as high heart rate, excessive sweating, muscle tension, shaking, dizziness, and fainting. The symptoms of depression considered include irritable mood or an overall low mood, sleep disorders (difficulty getting to sleep or excessive sleeping), changes in appetite (often with associated weight changes), tiredness and low energy, feelings of uselessness, self-hate and guilt, difficulty concentrating, slow or fast movements, feelings of hopelessness or abandonment, repetitive thoughts regarding death or suicidal thinking, and loss of pleasure in things that normally bring joy, including sexual activity.

The main researcher evaluated each questionnaire and those which did not fulfill the three requirements (>18 years of age, confirmed diagnosis of a rare disease, and no recent history of anxiety or depression) were excluded from the study.

Patients who failed to complete more than 85% of the questionnaire were also excluded.

### 2.2. Instruments

Depression was measured by the Patient Health Questionnaire (PHQ-9), an instrument that scores 9 items that determine the level of severity of depressive symptoms [13,14,15].

Cut-off points of a total score of 5, 10, 15, and 20 represent mild, moderate, moderately severe, and severe depression, respectively. A score of 10 or above is recommended by the authors as a single cut-off point for major depression. The instrument has an excellent internal reliability *(α* = 0.89), excellent test–retest reliability, and criterion and construct validity [13].

Anxiety was evaluated by the General Anxiety Disorder scale (GAD-7) [15,16,17]. It consists of seven questions that can be scored between 0 and 3; therefore, the minimum and maximum scores possible are 0 and 21, respectively. To evaluate the results obtained from the answered questionnaires, its authors suggest the following scores: 0–4, no anxiety detected, 5–9, symptoms of mild anxiety detected, 10–14, symptoms of moderate anxiety detected, and 15–21, symptoms of severe anxiety detected. It is recommended that consultation with a healthcare professional should be sought if a score of 10 or above is obtained.

Although it has the best operational characteristics for the detection of general anxiety disorder, the instrument has proven to be useful in the detection of any other anxiety disorder. The GAD–7 scale presents an excellent internal reliability (*α* = 0.92), good test–retest reliability (*ICC* = 0.83) as well as criterion, construct, factorial, and procedural validity.

### 2.3. Statistical Analyzsis

Mean and standard deviation were calculated for quantitative variables. Qualitative variables were reported using frequencies.

The Kolmogorov–Smirnov test was applied to test for normal distribution of the variables PHQ-9 score and GAD-7 score; of the two, the PHQ-9 could be considered normal (*p* = 0.11), a skewness of 0.96 and a kurtosis of 0.33.

Student’s *t*–test was used to determine if there were differences between PHQ-9 and GAD-7 scores according to whether the participant had been ill with COVID-19, had been in close contact with a COVID-19 patient, had non-cohabiting family members or friends who had been ill with COVID-19, had family members or close friends pass away due to COVID-19, age, gender, education level, marital status, and body mass index.

The relationship between quantitative and categorical variables was measured using the chi square test.

The relation between depression and anxiety was calculated applying Pearson’s correlation coefficient.

## 3. Results

To assess the reliability of the questionnaires, Cronbach’s alpha coefficient was calculated. For the questionnaire that measures depression, the Patient Health Questionnaire (PHQ-9), the Cronbach’s alpha coefficient was 0.90, whereas for the questionnaire that measures anxiety, the General Anxiety Disorder scale (GAD-7), the coefficient was 0.94. Overall, when all items were considered, the result was 0.95.

We analyzed a sample of 86 individuals, 76% of whom were female, and the mean age of the study group was 42.74 years (*SD* = 13.43). The majority of those surveyed, 95% (82 patients), lived in Spain, but there were also participants from Chile, Colombia, Holland and Mexico. Nearly one third of the participants (33%) were not in paid employment, either due to retirement or to recognition of permanent disability.

More than half of the participants, 57%, were married, followed by 33% who were single. Most of the participants in the study (54%) had university level education, an additional 9% had upper level professional training, and 14% had baccalaureate or middle level training.

At the time of reporting, 89% did not use tobacco and only 13% drank alcohol regularly.

Six participants (7%) had been ill with COVID-19, all of whom were symptomatic, and none required hospital admission. Regarding the symptoms experienced by the participants who had COVID-19, the highest percentages were found for dry cough and tiredness, followed by muscular pain and fever.

A minimum ten-day quarantine had to be completed by 21% (18 cases) of participants due to having close contact with someone who had COVID-19, and 45% (39 cases) had family members or close friends who had COVID-19 but did not have to be quarantined. We found that 14% (12 cases) had lost at least one family member or close friend due to COVID-19.

Analyzing the responses to the GAD-7 questionnaire, we observed no statistically significant differences between sexes *χ*^2^(3, *N* = 86) = 0.21, *p* = 0.98, and found that 74% did not have symptoms of anxiety or had mild symptoms (Table 1). Examining the answers according to education level, we found that among those with severe anxiety, there was a majority of individuals with university degrees (70%).

Regarding level of depression according to the score obtained from the PHQ-9 questionnaire, we found that 70% of the participants did not have symptoms of depression, or such symptoms were mild or very mild. Only 16% (14 individuals) had moderately severe or severe symptoms. The PHQ-9 scores were not associated to sex *χ*^2^(4, *n* = 86) = 2.24, *p* = 0.69 (Table 2).

We found no statistically significant differences according to body mass index, nor according to age in GAD-7 and PHQ-9 questionnaire scores.

There was a strong correlation *r*(86) = 0.75, *p* < 0.001, between the GAD-7 and PHQ-9 questionnaire scores, indicating that those who had the highest levels of anxiety were also likely to have high levels of depression.

Table 3 shows the mean and standard deviations according to the answers to the questions regarding COVID-19. Additionally, we used the *t* test to calculate the *p* value for the comparison of the groups formed according to answer.

As shown in Table 3, there were only differences in PHQ-9 scores amongst those who had family members or close friends who had been ill with COVID-19 and those who did not. The highest score was found in those who had family members or friends who had COVID-19. We found no differences in scores regarding sex, marital status or education level.

## 4. Discussion

This research studied the impact on health and psychological wellbeing in patients with rare diseases during the COVID-19 pandemic. More specifically, it focused on the evaluation of the degree of anxiety and/or depression that patients with rare diseases may have experienced during the pandemic. Patients with rare diseases are patients with high requirements of healthcare; however, throughout the pandemic, access to healthcare services has been limited and, in many cases, non-existent [18].

We have not found specific studies evaluating depression and anxiety in patients with rare diseases during the pandemic. During our literature search, we only retrieved one study, published prior to the pandemic, that focused on the same area of research as the present study [19]. For this reason, we reviewed and used the literature on rare diseases in general and specific rare diseases, and on chronic and rare diseases in which depression and anxiety have been studied. We do not disregard the possibility of pursuing new future lines of research to study all kinds of factors that affect anxiety and depression as well as sexual disfunction in patients with rare diseases [15,20].

In order to evaluate the depression parameter, we used the validated PHQ-9 questionnaire, which has been proven to be precise in the evaluation of depressive states [21]. This questionnaire has been evaluated and has been proven to have good psychometric properties. Therefore, it is considered an exact diagnostic tool for depressive states [22], and it has been utilized by other authors to evaluate depression in patients with rare diseases [4]. The contrast of results with those obtained in similar circumstances using the same tools is therefore possible.

Our results indicate that 70% of the patients considered did not show signs of depression or had some form of mild depression. Meanwhile, only 30% reported levels of moderately severe depression. We also demonstrated that the highest percentages of moderate to severe depression are found in female patients (34% of patients within same sex) compared to male patients (20%). In the study on depression and anxiety in patients with rare diseases published in 2019 [19], the results show that 42% of patients had symptoms of moderate or severe depression compared to the 34% found in this study. However, in other studies that focused on depression and anxiety during the pandemic [23,24] in the general population, rates of depression were at 16% [24] and 19% [23], respectively. In a comparison of the prevalence of depression in the general population during the pandemic and the prevalence in patients with rare diseases, our study found that the prevalence of depression in patients with rare diseases was nearly double that of the general population (30%).

For the study and evaluation of anxiety, we used the validated GAD-7 questionnaire. This questionnaire has been used in previous studies with the same purpose [19,23]. The data obtained in our study indicated that 26% of the patients considered had severe anxiety compared to 74% who had mild anxiety or no symptoms of anxiety. In the previously mentioned study in patients with rare diseases published prior to the pandemic [19], researchers found that 23% of patients had anxiety. In studies carried out in the general population during the pandemic, the results show percentages of moderate to severe anxiety of 14% [22] and 29% [23], respectively.

In the systematic review carried out by Xiong et al. [25], where they analyzed 19 articles that considered anxiety and depression in the general population during the pandemic, rates of anxiety ranged from 6% to 19%. The results obtained in our study of patients with rare diseases were higher, with the prevalence of depression ranging between 15% and 48%. These results for depression do not constitute a significant difference between the general population group and the results of this study. In their study, Xiong et al. also demonstrated that women had a higher rate of symptoms of depression than men [25], as did other studies [23] on depression and anxiety in the general population. The age group most affected was those younger than 40 years of age, and in our study, we found no relevance in the prevalence according to age.

On the other hand, there is a study on the impact of the pandemic in patients with rare diseases that demonstrates that 66% of the patients included in the study found their health care affected in some way during the pandemic. Around 46% of the study population suffered direct effects on their health status and wellbeing. Additionally, 79% reported that the pandemic had affected their mental health [25]. We did not find any articles including situational parameters regarding COVID-19 (Have you been ill with COVID-19? Have you been a close contact? etc.) such as those considered in this study in relation to anxiety and depression in patients with rare diseases.

In this study, we demonstrated that those patients who had family members or friends who were ill with COVID-19, even if they did not live together, had more symptoms of anxiety and depression than those who had been ill themselves or had been a close contact (*p* = 0.02). We did not find other published studies that relate depression and anxiety parameters with the patient’s situation regarding COVID-19 in order to contrast this fact.

This study was performed using an online cross-sectional design, the method of preference for other similar studies [19,26,27]. Among the weaknesses of cross-sectional studies is the inability to determine the temporal relation between outcomes and risk factors, so they are unable to establish if the exposure preceded the disease or vice versa. Therefore, in our study, it is not possible to know if the symptoms of depression or anxiety were present prior to the pandemic or arose due such circumstances.

Our sample included 86 patients, which constitutes a high “n” for the type of patients considered. Other studies on patients with rare diseases have been performed in as few as 15 patients [26]. Recruitment of this sample was carried out online, using online forms for data collection, due to the state of emergency decreed by the national government. This method has been used by other authors, including Ozamiz-Etxebarria [28], to collect information during the pandemic period. The main advantages of this method, compared to previous research methods, include privacy and timely management and access to the results. This study included a patient support system for study participants, so patients felt supported and were able to receive answers to their queries through an email account specifically created for that purpose. We found no evidence of a similar channel being used in other studies. Another interesting fact worth mentioning is the predominant female sex in this study, as in others carried out among patients with rare diseases [19].

## 5. Conclusions

Patients with rare diseases are a population group who have high healthcare requirements, and health services have been deeply affected by the pandemic. Therefore, health status and psychological wellbeing of these patients have also been affected.

As the results presented above suggest, levels of depression and anxiety may have been influenced by the current pandemic state as levels of moderate or severe anxiety were higher in patients with rare diseases than in those with other types of chronic diseases and the general population, as it is found in 26% of the study population. Additionally, the percentage of patients with moderate or severe depression (30%) among patients with rare diseases during the pandemic nearly doubled the percentage of those affected in the general population during the same period. However, comparing the results to those of a 2019 study in patients with rare diseases, the levels remained at similar levels prior to and during the pandemic. This study found that symptoms such as anxiety and depression have a higher prevalence among women than men. The uncertainty surrounding the pandemic may increase anxiety and depression, as the patients with higher levels of symptoms are those who had family members or close friends who had been ill or passed away due to COVID-19, more so than those who were ill themselves. The study results indicate that during the pandemic, anxiety/depression remained at a higher level in this group than in the general population.

Depression and anxiety in patients with rare diseases is an issue that needs further research, as there are not many publications that consider rare diseases as a whole. There are several articles on certain specific diseases, but none including patients with rare diseases in general.

## Figures and Tables

**Table 1:** General Anxiety Disorder scale (GAD-7) questionnaire scores according to sex of the studied population.

		No	Mild	Moderate	Severe	Total
Women	*n*	20	28	9	8	65
	% within sex	31	43	14	12	100
	% within GAD-7	77	74	75	80	76
Men	*n*	6	10	3	2	21
	% within sex	29	48	14	9	100
	% within GAD-7	23	26	25	20	24
Total	*n*	26	38	12	10	86
	% within sex	30	44	14	12	100
	% within GAD-7	100	100	100	100	100

**Table 2:** Patient Health Questionnaire (PHQ-9) scores according to sex of the studied population.

		No	Mild	Moderate	Moderately Severe	Severe	Total
Women	*n*	22	21	10	7	5	65
	% within sex	34	32	15	11	8	100
	% within GAD-7	76	68	83	87	83	76
Men	*n*	7	10	2	1	1	21
	% within sex	33	48	9	5	5	100
	% within GAD-7	24	32	17	12	17	24
Total	*n*	29	31	12	8	6	86
	% within sex	34	36	14	9	7	100
	% within GAD-7	100	100	100	100	100	100

**Table 3:** Comparison of means and standard deviation according to answers by PHQ-9 and GAD-7 scores.

		YES	NO	P
Have you been ill with COVID-19?	PHQ–9	7.50 (5.28)	7.81 (6.46)	0.91
	GAD–7	6.67 (3.39)	7.36 (5.57)	0.76
If you did not have COVID-19, have you been a close contact of a COVID-19 patient?	PHQ–9	6.83 (6.18)	8.04 (6.42)	0.48
	GAD–7	7.39 (5.49)	7.29 (5.46)	0.95
Have any of your non cohabiting family members or close friends been ill with COVID-19?	PHQ–9	9.49 (6.30)	6.38 (6.12)	0.02
	GAD–7	7.82 (5.66)	6.89 (5.26)	0.43
Have any of your family members or close friends passed away due to COVID-19?	PHQ–9	10.33 (7.30)	7.38 (6.14)	0.14
	GAD–7	9.58 (6.68)	6.95 (5.16)	0.12

## Supplementary Materials

Participants accessed and completed the questionnaires through the online form link: https://docs.google.com/forms/d/e/1FAIpQLSdJ2woR6VTxl7LRt8-B1uTBrvHMaF0_mChMMhxqsi2bYWiw9A/viewform?usp=sf_link.
